# Supplementary material for: Resolving the full spectrum of human genome variation using Linked-Reads
Source: Genome Res. 2019 Apr;29(4):635–45. doi: 10.1101/gr.234443.118 (PMC6442396; doi:10.1101/gr.234443.118)
Supplement: Supplemental Material [file supp_gr.234443.118_Supplemental_longranger-2.2.zip › longranger-2.2.tar.bz2!/lr2.2-public/lib/stan/cmdstan/stan/doc/api/html/write__stan_8hpp_source.html]

Stan: src/stan/common/write\_stan.hpp Source File


|  |  |
| --- | --- |
|  | Stan  2.5.0  probability, sampling & optimization |


- Main Page
- Related Pages
- Namespaces
- Classes
- Files

- File List
- File Members

All Classes Namespaces Files Functions Variables Typedefs Enumerations Enumerator Friends Macros Pages

- src
- stan
- common

write\_stan.hpp

Go to the documentation of this file.

1 #ifndef STAN\_\_COMMON\_\_WRITE\_STAN\_HPP

2 #define STAN\_\_COMMON\_\_WRITE\_STAN\_HPP

3

4 #include <ostream>

5 #include <string>

6 #include <stan/version.hpp>

7

8 namespace stan {

9

10  namespace common {

11

12  void write\_stan(std::ostream\* s, const std::string prefix = "") {

13  if (!s) return;

14

15  \*s << prefix << " stan\_version\_major = " << stan::MAJOR\_VERSION << std::endl;

16  \*s << prefix << " stan\_version\_minor = " << stan::MINOR\_VERSION << std::endl;

17  \*s << prefix << " stan\_version\_patch = " << stan::PATCH\_VERSION << std::endl;

18  }

19

20  } // namespace common

21

22 } // namespace stan

23

24 #endif

stan::common::write\_stan

void write\_stan(std::ostream \*s, const std::string prefix="")

**Definition:** write\_stan.hpp:12

stan::MINOR\_VERSION

const std::string MINOR\_VERSION

Minor version number for Stan package.

**Definition:** version.hpp:12

stan::PATCH\_VERSION

const std::string PATCH\_VERSION

Patch version for Stan package.

**Definition:** version.hpp:15

stan::MAJOR\_VERSION

const std::string MAJOR\_VERSION

Major version number for Stan package.

**Definition:** version.hpp:9

version.hpp


---

[ Stan Home Page ]

*© 2011–2014,
Stan Development Team.*
